# *S100A8* expression in oviduct mucosal epithelial cells is regulated by estrogen and affects mucosal immune homeostasis

**DOI:** 10.1371/journal.pone.0260188

**Published:** 2021-11-18

**Authors:** Xiaodan Li, Guifang Cao, Hongxin Yang, Dafu Zhi, Lei Li, Daqing Wang, Moning Liu, Hong Su

**Affiliations:** 1 Inner Mongolia Key Laboratory of Basic Veterinary Science, Inner Mongolia Agricultural University, Hohhot, China; 2 Department of Basic Medicine, Inner Mongolia Medical University, Hohhot, China; 3 Shenzhen Institute of Advanced Technology, Chinese Academy of Sciences, Shenzhen, China; 4 Maternal and Child Health Hospital of Hohhot, Hohhot, China; 5 Inner Mongolia Academy of Agriculture and Animal Husbandry Sciences, Hohhot, China; Icahn School of Medicine at Mount Sinai, UNITED STATES

## Abstract

Chronic inflammation can cause oviduct mucosal damage and immune dysfunction, leading to infertility, early pregnancy loss, ectopic pregnancy, tumors, and a decrease in reproductive capacities in female animals. Estrogen can suppress immune responses in different tissues and oviducts, and regulate the oviduct immune balance; however, the underlying mechanisms remain unclear. The objective of this study was to explore the mechanism of estrogen-regulated oviduct mucosal immunity and discover new estrogen targets for regulating oviduct mucosal immune homeostasis. Sheep oviduct epithelial cells (SOECs) were treated with 17-β estradiol (E2). Transcriptome sequencing and analysis showed differentially expressed S100 calcium-binding protein A (*S100A*) genes that may participate in the oviduct mucosa immunoregulation of estrogen. Quantitative polymerase chain reaction and immunocytochemistry analysis showed that *S100A8* expression changed dynamically in E2-treated SOECs and peaked after 7 h of treatment. Estrogen nuclear receptors and G protein-coupled membrane receptors promoted E2-dependent *S100A8* upregulation. The *S100A8* gene was disrupted using the clustered regularly interspaced short palindromic repeats (CRISPR)/CRISPR-associated protein 9 method. Levels of inflammatory factors interleukin (IL)-1β and IL-4 were significantly upregulated in *S100A8*-knockdown SOECs, whereas those of the anti-inflammatory factor IL-10 was downregulated. Following S100A8 knockdown in SOECs treated with E2 for 7 h, IL-10 levels increased significantly. Estrogen affected oviduct mucosa immune function and dynamically regulated S100A8 in SOECs. S100A8 knockdown caused an excessive immune response, indicating that S100A8 is beneficial for maintaining immune homeostasis in the oviduct mucosa. Moreover, estrogen can compensate for the effect of S100A8 knockdown by upregulating IL-10.

## Introduction

The oviduct is an important component of the female reproductive system and serves as the site of gamete transport, fertilization, and early embryo development. The function of the oviduct in reproduction has received increasing attention in recent years [1]. The oviduct mucosa maintains the immune environment inside the lumen, but chronic inflammation can cause oviduct mucosal damage and immune dysfunction, which can lead to infertility, early pregnancy abortion, ectopic pregnancy, and even tumors, as well as decreased production and reproductive capacities of female animals [2–5]. Acute inflammation of the fallopian tube can be treated with antibiotics or surgery; however, antibiotics, dexamethasone, and non-steroidal anti-inflammatory drugs have little effect on mucosal damage and immune dysfunction caused by persistent chronic inflammation [6,7].

Estrogen can inhibit the inflammatory responses of different tissues under various circumstances, such as during hepatitis, vaginitis, nerve damage, or respiratory tract infection [8–12]. In bovine oviducts, estrogen regulates the balance of T helper type 1 (Th1)/Th2 immune responses; it also exerts a suppressive effect on the function of Toll-like receptor 3 in the fallopian tube cell [13,14]. Estrogen receptor-α (ER-α) inhibits the innate immune response in mouse oviducts by regulating innate immune mediators and preventing embryo death [15]. The downregulation of estrogen and progesterone is well correlated with the increased expression of cytokines in the chicken-molting period [16]. These studies show that estrogen regulates inflammation and immune dysfunction in oviducts, although the understanding of the mechanism whereby estrogen regulates oviduct mucosal immunity is still limited.

Thus, the aim of this study was to explore the mechanism of estrogen-regulated oviduct mucosal immunity and discover new estrogen targets for regulating oviduct mucosal immune homeostasis. Exploring the mechanism whereby estrogen regulates the immune homeostasis of the oviduct mucosa can provide a reference for estrogen therapy. As estrogen therapy currently poses potential risks [17–19], the discovery of new estrogen targets for regulating the immune function of the oviduct mucosa may be helpful for reducing oviduct mucosal damage caused by continuous inflammation and correcting oviduct diseases.

## Materials and methods

### Obtaining oviducts

The handling and euthanasia of the experimental animals were approved by the Special Committee on Scientific Research and Academic Ethics of Inner Mongolia Agricultural University (approval number: [2020]008). The animal protocols were implemented in accordance with the “*Guidelines for Euthanasia of Experimental Animals T/CALAS 31–2017*” (issued by the Chinese Association for Laboratory Animal Science) and the “*Guide for the Care and Use of Laboratory Animals*” (published by the National Academy of Sciences, The National Academies Press, Washington, D.C.). The animals purchased from the farm (Siheyi Village, Wuchuan County, Hohhot) and were euthanized by injecting an overdose of barbiturates intravenously, the fallopian tubes were removed after bleeding.

The animals used in this study were interestrus, sexually mature (12–15 months), and robust female, *small-tailed Han* sheep. The oviducts were removed from the abdominal cavity and stored in a bacteria-free sample bottle containing penicillin–streptomycin in phosphate-buffered saline.

### Culturing and treating sheep oviduct epithelial cells (SOECs)

The oviduct ampullae were cut longitudinally, and the mucosal epithelial cells were scraped after trypsin digestion as described by Wen *et al*. [20,21]. All cells used in the experiments were at passage number three.

The cells were starved and cultured in serum-free and phenol red-free medium for 12 h to reduce any effects of serum and phenol red. The cultured SOECs were treated with 17β-estradiol (E2) at a final concentration of 1 × 10^−8^ M [20,22], and samples were taken for RNA-sequencing (RNA-seq) after E2 treatment for 0 (T0 control), 1.5 (T1 group), 3.5 (T2 group), and 5.5 h (T3 group). Samples were obtained for quantitative polymerase chain reaction (q-PCR) analysis after E2 treatment for 0 (control), 2, 4, or 6 h. The experiments were repeated three times, and total RNA was used for transcriptome sequencing and q-PCR validation.

Based on the RNA-seq and q-PCR-validation results, we further verified whether S100 calcium-binding protein A8 (*S100A8*) mRNA expression continued to increase after E2 treatment at longer time points. To this end, the SOECs were treated with 10^−8^ M E2 and divided into 5, 6, 7, and 8 h-treatment groups and a control group (0 h). After determining when S100A8 expression peaked following E2 treatment, that time point was selected and the cells were treated with 10^−6^, 10^−7^, 10^−8^, or 10^−9^ M E2 [23–25]. An untreated control group was also tested. q-PCR was performed to detect *S100A8* expression. Based on these results, SOECs were exposed to 10^−8^ M E2 for 7 h, and immunofluorescence was performed to detect the *S100A8* protein expression in SOECs compared with that in the control group. The experiments were repeated three times.

To verify that the estrogen receptor upregulated *S100A8* mRNA expression, SOECs were treated with three different estrogen action inhibitors (EAIs), namely fulvestrant, tamoxifen, and G-15. The cells were grouped into fulvestrant (10^−9^ M) + E2, tamoxifen (10^−7^ M) + E2, G-15 (10^−7^ M) + E2, tamoxifen + G-15 + E2, fulvestrant + G-15 + E2, -E2, and E2 (control) groups. The -E2 group was not treated with E2 or any inhibitor, whereas the E2 group was treated with E2, but no inhibitor. After adding different inhibitors to each experimental group for 0.5 h, the groups were treated with 10^−8^ M E2 for 7 h. q-PCR and western blot (WB) analyses were performed to detect *S100A8* expression levels in triplicate independent experiments.

### Transcriptome sequencing

Valid data were obtained after quality evaluation. Adaptor reads, low-quality reads, and reads that contained more than 5% N (N refers to base information that could not be determined) were removed from the original data. Based on the Ensemble Genome and mRNA database, TopHat2 software (version 2.0.9) was used to compare a reference genome with the preprocessed valid data. Based on the comparison results, the Cufflinks series program (version 2.1.1) was used to splice and annotate the sequence fragments and calculate the transcript expression levels. The fragments per kilobase of transcript per million mapped reads (FPKM) method is used to represent the expression abundances of known genes in different samples, and an FPKM value of >0 was used as the detection standard for genes in this study. The threshold of highly altered genes (HAGs) was set to P < 0.05. HAGs were analyzed using the Gene Ontology (GO) knowledgebase for functional enrichment analysis and Kyoto Encyclopedia of Genes and Genomes (KEGG) orthology-based annotation system for signaling pathway-enrichment analysis.

### Real-time q-PCR analysis

The mRNA expression levels of five HAGs, namely, *S100A8*, *S100A9*, *S100A12*, C-X-C motif chemokine receptor 4 (*CXCR4*), and cytochrome P450 1B1 (*CYP1B1*), were determined by q-PCR (CFX96 System, Bio-Rad, USA). NCBI-Primer BLAST was used to design the primers shown in Table 1, and relative mRNA expression levels were calculated using the 2^-ΔΔCT^ method, with *β-Actin* mRNA serving as an endogenous reference. The RNA-seq results were verified based on the changing trends of the above five genes.

**Table 1:** Primer sequences of five genes.

Gene		Primer sequence (5’-3’)
***S100A8***	Forward primer	GTGGGGCAAATCCTTGGACA
	Reverse primer	TGAACCAAGTGTCCGCATCC
***S100A9***	Forward primer	CGGAAACCCTGATCCGGAAA
	Reverse primer	CCTGGCCACCAGCATAATGA
***S100A12***	Forward primer	TACGACACCCTCGTCAAGTG
	Reverse primer	TGGACACCAGGACTACGAAC
***CXCR4***	Forward primer	ACTGAGGATGACTTGGGCTC
	Reverse primer	TGTCCGTCATGCTCCTTAGC
***CYP1B1***	Forward primer	GTGGCTGCTCGTTCTCTTCA
	Reverse primer	GGAATGGTGAGGGGCACAAA
***β-actin***	Forward primer	GTCACCAACTGGGACGACA
	Reverse primer	AGGCGTACAGGGACAGC

We used q-PCR to study E2-dependent regulation of *S100A8* expression in SOECs at different times and E2 concentrations, the effect of EAIs on *S100A8* expression, and the knockdown efficiency of the *S100A8* gene. The *S100A8* primers, endogenous reference, and calculation method are described above.

### Immunofluorescence detection method

SOECs were fixed in acetone, treated with Triton-X 100, and labeled with a primary anti-S100A8 antibody (1:1000, rabbit anti-MRP8 polyclonal antibody, catalogue no. ab180735, Abcam, UK) and a secondary antibody (1:200, goat anti-rabbit IgG/FITC antibody, catalogue no. bs-0295G-FITC, Bioss, China). A laser-scanning confocal microscope (Nikon, Japan) was used to take images of the SOECs.

### Knocking out the *S100A8* gene

**Fig 1:**
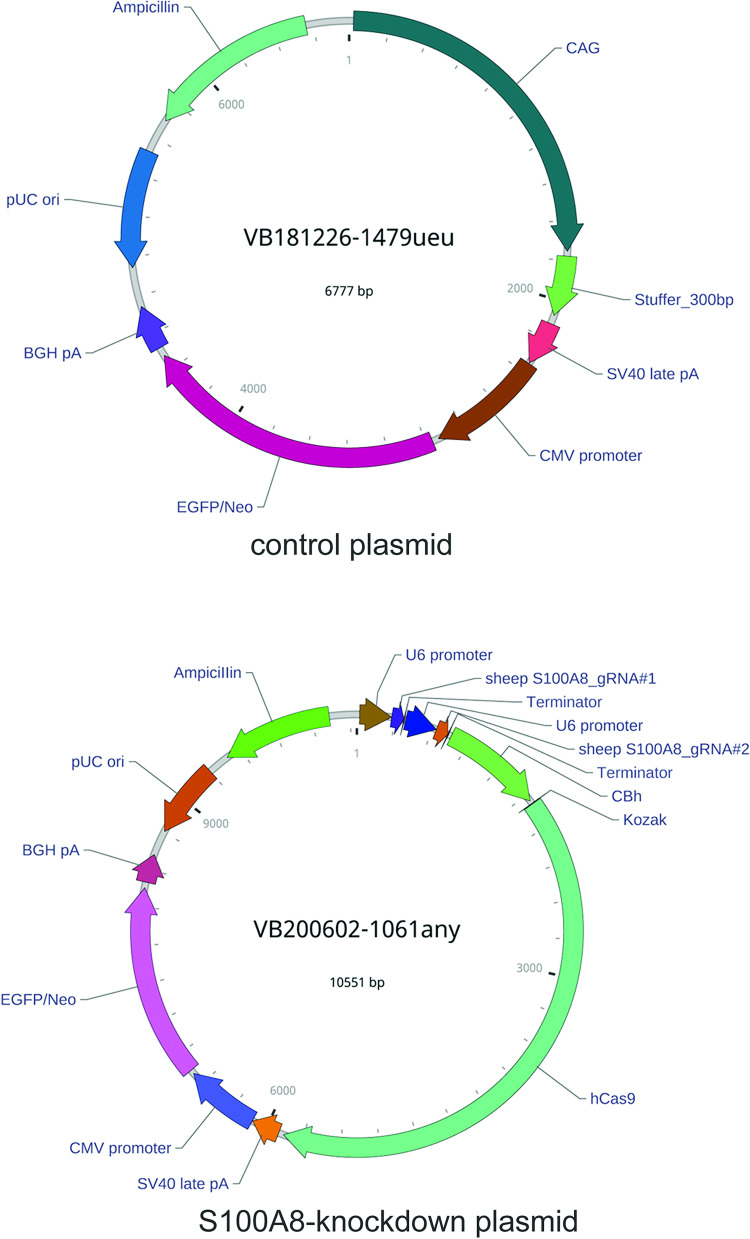
The control and the S100A8-knockout plasmid vectors.

The clustered regularly interspaced short palindromic repeats (CRISPR)/CRISPR-associated protein 9 (Cas9) gene knockout method was used to explore the possible impact of S100A8 on immune homeostasis of the oviduct mucosa. Guide RNA (gRNA) was designed at http://crispr.mit.edu/database based on the sheep *S100A8* sequence deposited in NCBI (Gene ID: 101104026). Two g-RNAs were designed to target different sites of the same gene (Table 2) and both gRNA sequences were cloned in tandem into a CRISPR/Cas9 vector (VB200602-1061any, Yunzhou Biotechnology Company, China). The vector was constructed using gateway technology, and a control plasmid (VB181226-1479ueu, Yunzhou Biotechnology Company, China) was used in the control experiments (Fig 1). DNA plasmids were extracted and purified (Endofree Plasmid Mini Kit, CW biotech, China) and transfected into cultured SOECs using a 1:3 ratio of plasmid DNA:transfection reagent (X-treme Gene HP DNA Transfection Reagent, Roche, USA). The control group was transfected similarly with a control plasmid, and the experiment was repeated three times. *S100A8* expression was assessed by q-PCR and WB method 48 h post-transfection.

**Table 2:** S100A8 gRNA sequences in CRISPR/Cas9 vector.

Name	Sequence of gRNA (5′→3′)
**sheep *S100A8*_ gRNA#1-F**	CTTCTCACCTTCAAAAACTTGTTTTAGAGCTAGAAATAGCAAGTTAAAATAAGGCTAGTCCGTTATCAACTTGAAAAAGTGGCACCGAGTCGGTGC
**sheep *S100A8*_ gRNA#1-R**	GCACCGACTCGGTGCCACTTTTTCAAGTTGATAACGGACTAGCCTTATTTTAACTTGCTATTTCTAGCTCTAAAACAAGTTTTTGAAGGTGAGAAG
**sheep *S100A8*_ gRNA#2-F**	GGATGGTGGAATTAACTTCGGTTTTAGAGCTAGAAATAGCAAGTTAAAATAAGGCTAGTCCGTTATCAACTTGAAAAAGTGGCACCGAGTCGGTGC
**sheep *S100A8*_ gRNA#2-R**	GCACCGACTCGGTGCCACTTTTTCAAGTTGATAACGGACTAGCCTTATTTTAACTTGCTATTTCTAGCTCTAAAACCGAAGTTAATTCCACCATCC

Note: F is the Forward strand, R is the Reverse strand.

### WB analysis

Total cellular proteins were extracted following different treatments. WB analysis was performed by first resolving the extracted proteins by sodium dodecyl sulfate-polyacrylamide gel electrophoresis, transferring them to a polyvinylidene membrane, and incubating the membrane with primary antibodies (1:1000, rabbit anti-MRP8 polyclonal antibody, catalogue no. ab180735, Abcam; 1:10000, rabbit anti-GAPDH monoclonal antibody, catalogue no. ab181603, Abcam) at 4°C for 12 h. Then, the membrane was incubated in a horseradish peroxidase-conjugated secondary antibody (1:6000, goat anti-rabbit IgG secondary antibody HRP conjugate, catalogue no. BA1054, Boster, China) at room temperature for 1.5 h. Subsequently, enhanced chemiluminescence was performed to visualize the bands. GAPDH was detected as a loading control. WB was performed to assess the effects of EAIs on S100A8 expression and evaluate the efficiency of S100A8 knockout using the CRISPR/Cas9 system.

### Enzyme-linked immunosorbent assay (ELISA) for testing cytokine levels

The cytokines interleukin (IL)-1β, tumor necrosis factor (TNF)-α, IL-4, and IL-10 in cell lysates were tested using ELISA kits (Sheep IL-1β/TNF-α/IL-4/IL-10 ELISA Kits, Meibiao, China). Optical density values were measured using a microplate reader (Eppendorf, Germany), and the cytokine content of the sample was calculated using a standard curve. Cytokine levels in the presence or absence of E2 were assessed by ELISA, both before and after S100A8 gene knockout.

### Statistical analysis

GraphPad Prism software (version 8.0) was used for drawing and statistical analysis of significant differences between groups. One-way or two-way analysis of variance was used to test significant for differences between groups (*p < 0.05, **p < 0.01). Image J software (version 1.34j) was used to measure the integrated densities of the WB images and normalize the protein expression level of the target gene with that of the loading control.

## Results and discussion

### Analysis of HAGs

**Fig 2:**
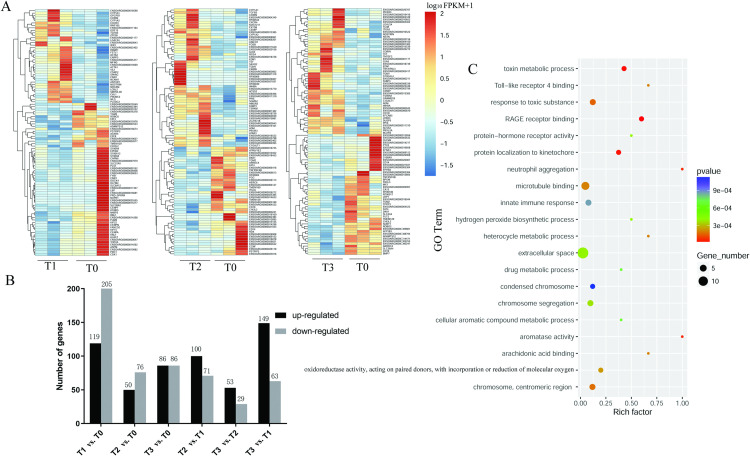
RNA-seq-based detection of HAGs in SOECs before (T0) and after E2 treatment for different times (T1/T2/T3) and functional analysis. (A) Cluster analysis of the top 100 HAGs between groups treated with E2 for different times and the control group (T1 vs. T0, T2 vs. T0, and T3 vs. T0; T1 = 1.5 h, T2 = 3.5 h, T3 = 5.5 h). (B) The number of HAGs between groups treated with E2 for different times and the control group. (C) Scatter plot of the top 20 GO terms associated with enriched HAGs (T1 vs. T0).

Cluster analysis showed the changes in HAG-expression levels at different times (T1/T2/T3) after E2 treatment compared with those before E2 treatment (T0). The results reflected the gene-regulation mode under estrogen treatment to a certain extent. The heatmap in Fig 2A shows the top 100 HAGs, expressed as the log_10_^(FPKM+1)^ values. We identified (i) 324 HAGs by comparing the T0 and T1 groups (119 upregulated genes and 205 downregulated genes); (ii) 126 HAGs by comparing the T0 and T2 groups (50 upregulated and 76 downregulated); (iii) 174 HAGs by comparing the T0 and T3 groups (86 upregulated and 86 downregulated); (iv) 171 HAGs by comparing the T2 and T1 groups (100 upregulated and 71 downregulated); and (v) 82 HAGs by comparing the T3 and T2 groups (53 upregulated and 29 downregulated). The HAGs showed dynamic changes over time after E2 treatment (Fig 2B).

GO-based functional analysis revealed that HAGs were significantly (p < 0.05) enriched in immune and inflammation-related functional terms, such as RAGE receptor binding, regulation of chemotaxis, natural immune response, and regulation of interferon-gamma production. These HAGs included *S100A8*, *S100A9*, *S100A12*, *CXCR4*, pentraxin 3 (*PTX3*), lipopolysaccharide-binding protein (*LBP*), and nuclear protein 1 (*NUPR1*). Cerny *et al*. found that S100A8- and S100A9-expression levels are correlated with ER-α in mice oviduct or breast cancer cells [26,27]. S100A8 is highly expressed in aging oviduct inflammation [28,29]. However, previous data show that, without affecting pathogen defense, S100A8 can regulate excessive inflammatory responses through myeloid differentiation primary response gene 88- or IL-10-dependent pathways, and that the role of S100A8 in inflammation is related to its polymeric conformation [30–35]. These imply that S100A8 may play an important role in regulating oviduct immune homeostasis by estrogen. In addition, GO analysis revealed that estrogen affected various physiological functions of SOECs (Fig 2C, Table 3).

**Table 3:** Some of the GO functions in which HAGs are significantly enriched (p < 0.05).

GO terms	HAGs enriched in GO terms
**Functions related to immune response and inflammation**	
Innate immune response; Regulation of chemotaxis; RAGE receptor binding; Negative regulation of immune response; Regulation of interferon-gamma production; Negative regulation of viral genome replication; Toll-like receptor 4 binding	*S100A9*, *S100A12*, *S100A8*, *CXCR4*, *PTX3*, *LBP*, *ISG17*, *MX1*, *OLFM4*
**Functions related to cell division and proliferation**	
Chromosome segregation; Positive regulation of cell proliferation protein localization to kinetochore chromosome; centromeric region	*CDC2*, *BUB1B*, *CASC5*, *CDCA8*, *CENPF*, *CENPN*, *MKI67*, *TOP2A*, *SKA1*, *FGF21*, *ADM*, *CCKBR*, *FGF18*, *FGFBP1*
**Functions related to differentiation**	
Epithelial cell differentiation; Positive regulation of epithelial cell differentiation; Cell morphogenesis involved in differentiation; Positive regulation of chondrocyte differentiation; Positive regulation of striated muscle cell differentiation	*VIL1*, *CDC2*, *PTHLH*, *DMBT1*, *DKK1*, *FGF18*, *HOPX*
**Functions related to development**	
Cell junction; Digestive tract development; Regulation of female gonad development; Oocyte development Inner ear development; Vascular smooth muscle cell development; Mitral valve development; Regulation of hair follicle development; Septum primum development	*ATP2B2*, *LGR5*, *GJA5*, *NUPR1*, *STRA8*, *CXCR4*, *DLG2*, *CCKBR*, *HEPS*, *ADM*
**Functions related to compound metabolism**	
Heterocycle metabolic process; Steroid metabolic process; Retinol metabolic process; Progesterone metabolic process; Arachidonic acid metabolic process	*CYP1A1*, *CYP1A2*, *CYP1B1*, *DHRS9*

Note: HAGs are highly altered genes between the E2 treatment groups and the control group (T1 vs. T0, T2 vs. T0, T3 vs. T0).

**Fig 3:**
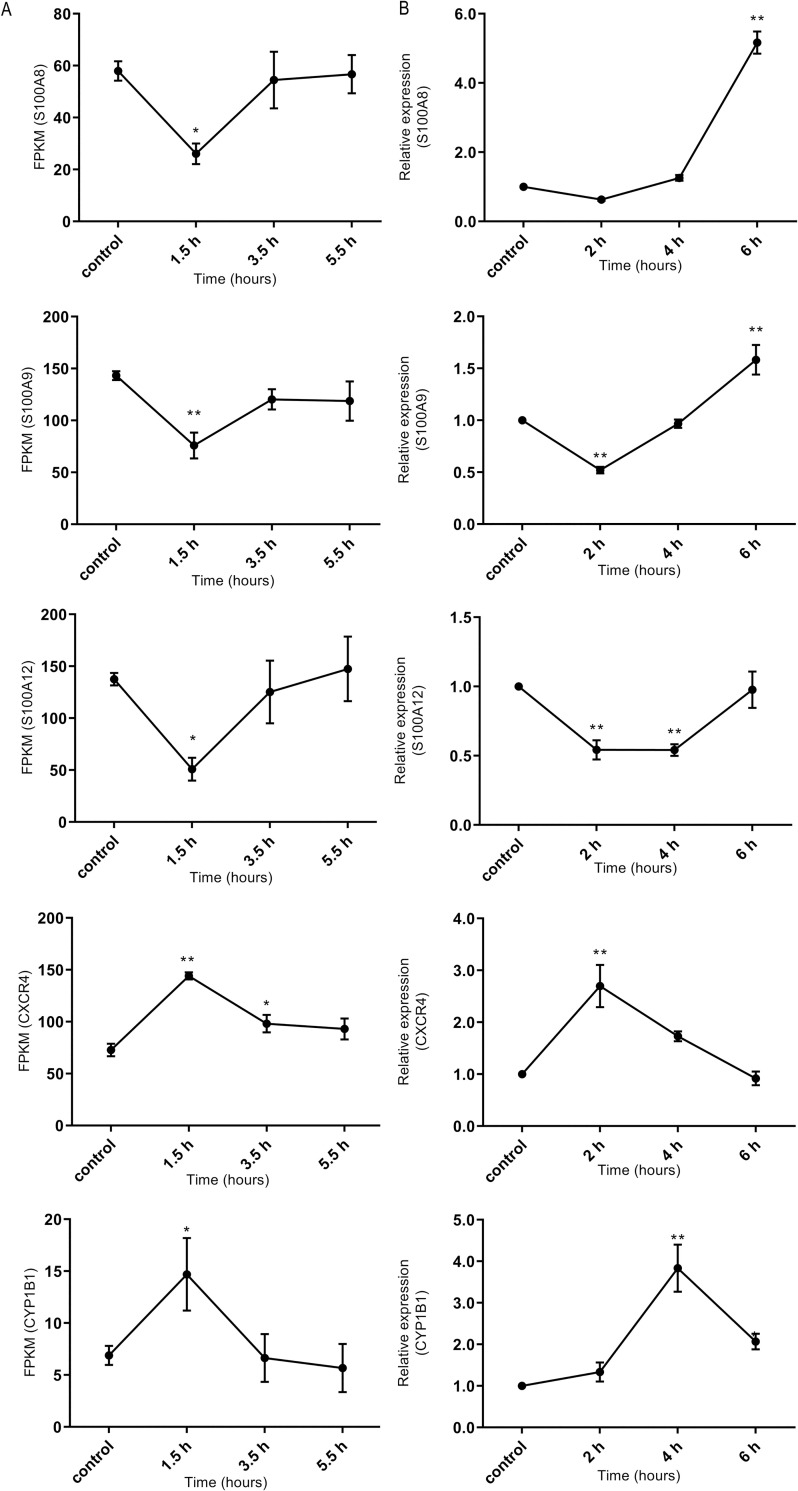
Expression change trends of five genes (*S100A8*, *S100A9*, *S100A12*, *CXCR4*, and *CYP1B1*) in SOECs following different treatment times with E2. (A) Expression change trends of genes with increasing E2-treatment times (0, 1.5, 3.5, and 5.5 h), as detected by RNA-seq. (B) Expression change trends of genes with increasing E2-treatment times (0, 2, 4, and 6 h), as verified by q-PCR. * (p < 0.05) and ** (p < 0.01) represent significant difference compared with the control group.

After E2 treatment for different times, the signaling pathways that continued to be affected in SOECs were represented with KEGG terms, such as chemokine signaling pathway, Toll-like receptor signaling pathway, arachidonic acid metabolism, leukocyte transendothelial migration, MAPK signaling pathway, calcium signaling pathway, regulation of actin cytoskeleton, retinol metabolism, steroid hormone biosynthesis, and progesterone-mediated oocyte maturation. The calcium signaling pathway, MAPK signaling pathway, Toll-like receptor signaling pathway, and arachidonic acid metabolism pathway are closely related to *S100A8* [36–38].

q-PCR verification showed that the expression change trends of five selected genes (*S100A8*, *S100A9*, *S100A12*, *CXCR4*, and *CYP1B1*) over time following E2 treatment were generally consistent with the RNA-seq results (Fig 3A and 3B), indicating the reliability of the RNA-seq data. The RNA-seq results showed that the expression levels of *S100A8*, *S100A9*, and *S100A12* tended to first decline and then rise after E2 treatment. However, to determine whether the expression levels returned to the baseline or continued to increase, it was necessary to delay the q-PCR analysis to later time points. We detected their gene expression levels in SOECs 2, 4, and 6 h after E2 treatment by q-PCR. The results showed that *S100A8* and *S100A9* mRNA levels increased significantly (p < 0.01) after 6 h of E2 treatment (Fig 3B).

### Involvement of ERs and G protein-coupled estrogen receptors (GPERs) in E2-dependent *S100A8* upregulation

**Fig 4:**
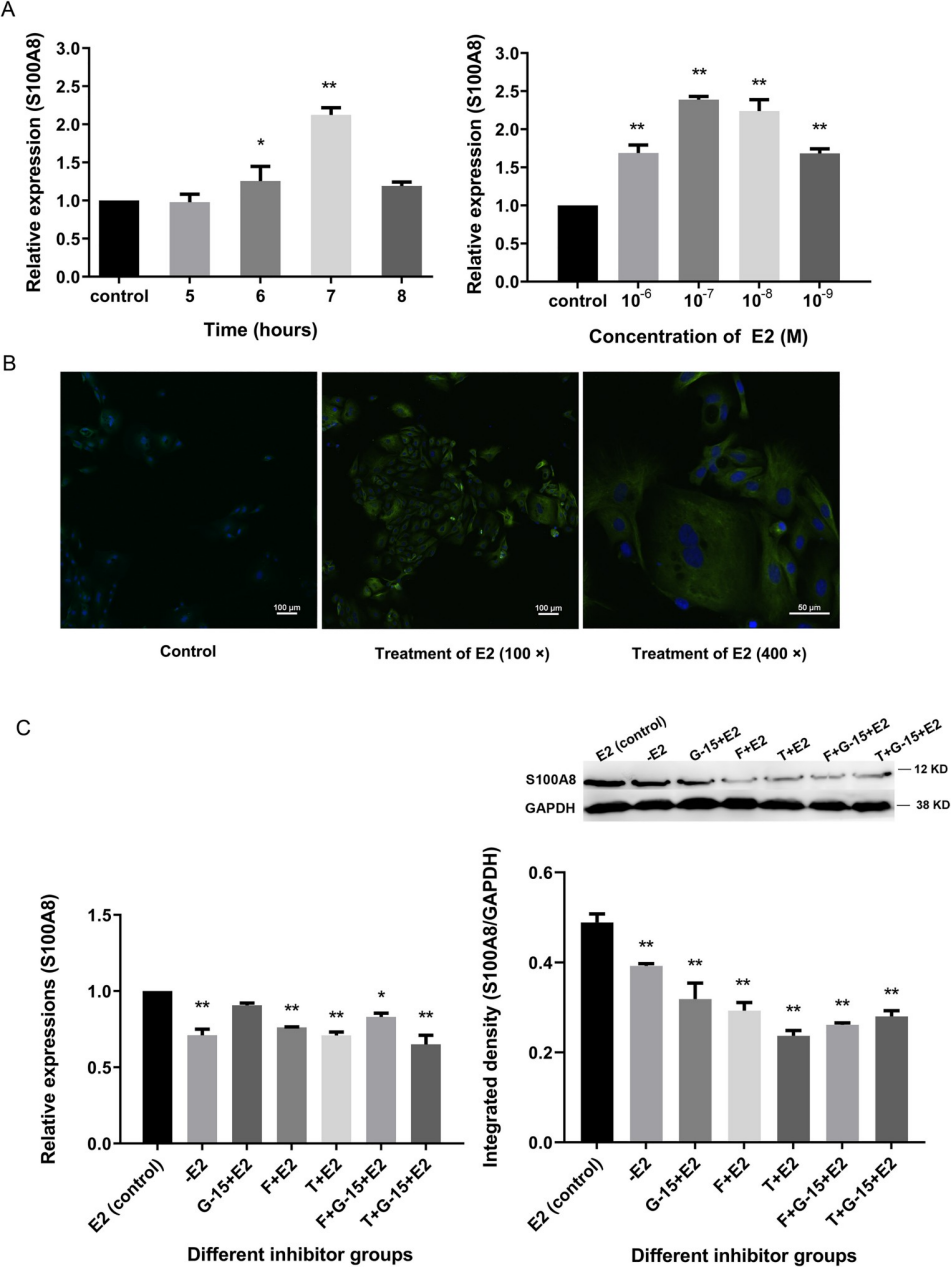
Effects of E2 and EAIs on *S100A8* expression in SOECs. (A) The relative mRNA expression levels of *S100A8* after different times (5, 6, 7, and 8 h) and different concentrations (10^−6^, 10^−7^, 10^−8^, and 10^−9^ M) of E2 treatment. The data represent the mean ± standard error (SEM) of three replicate experiments (n = 3). All values are normalized to β-actin expression. (B) Immunofluorescent detection of *S100A8* before (control) or after treatment with E2 (10^−8^ M) for 7 h. The target proteins are indicated with green fluorescence, and nuclei are indicated with blue fluorescence. The scale bars represent 100 μm or 50 μm. (C) Effects of three EAIs (G-15: 10^−7^ M, fulvestrant: 10^−9^ M, tamoxifen: 10^−7^ M) or combinations thereof on E2-dependent *S100A8* upregulation in SOECs. *S100A8* expression was tested in different groups by q-PCR and WB. The data represent the mean ± SEM of three replicate experiments. The values shown are normalized to *GAPDH* expression. * (p < 0.05) and ** (p < 0.01) represent significant difference compared with the control group.

As mentioned above, *S100A8* expression was dynamically regulated by E2, and it was increased significantly after 6 h of treatment. We performed q-PCR to further detect the mRNA expression levels of *S100A8* in SOECs treated with 10^−8^ M E2 for 5–8 h (Fig 4A). *S100A8* mRNA expression increased after 6 h of E2 treatment, then increased significantly (p < 0.01; versus the control group) and peaked after 7 h of treatment, and finally began to decrease after 8 h of treatment.

After 7 h (when *S100A8* expression peaked) of exposure to different concentrations of E2, the mRNA expression level of *S100A8* in the 10^−7^ M group was significantly (p < 0.01) higher than that in the control group and was the highest among all tested groups. The *S100A8* expression levels did not differ significantly between the 10^−7^ M and 10^−8^ M groups. The *S100A8* mRNA expression levels in the 10^−6^ M and 10^−9^ M groups were lower but were still significantly higher (p < 0.01) than those in the control group (Fig 4A). These findings showed that different E2 concentrations could increase *S100A8* expression after 7 h, but that *S100A8* mRNA expression was highest during E2 treatment at concentrations of 10^−7^ M and 10^−8^ M.

When SOECs were treated with 10^−8^ M E2 for 7 h, the immunofluorescent signals of S100A8 proteins were stronger than those in the control group, indicating that *S100A8* expression increased after treatment with 10^−8^ M E2 for 7 h, which is consistent with the q-PCR results (Fig 4B). Previous studies suggest that estrogen and ER have a positive regulatory effect on *S100A* protein. The *S100A8* and *S100A9* expressions in breast cancer tissues are down-regulated by the estrogen receptor antagonist tamoxifen, and the *S100A9* expression in ER+ tissues is significantly higher than that in ER- tissues [39]. *S100A8* expression increases in human blood during the proliferative phase of the menstrual cycle (increased estrogen), but it has been proposed that the increase in *S100A8* expression is related to the loss of early pregnancy [40]. However, *S100A8* and *S100A12* are more highly expressed in the endometrium of healthy cattle (versus postpartum cattle with uterine inflammation), suggesting that they may be more conducive to the health of the uterus and may prevent the maternal rejection of embryos [41–43].

Estrogen induces effects in cells through the non-genomic GPER pathway and the genomic nuclear ER pathway, and both independent pathways may converge by regulating gene transcription [44,45]. G-15 is considered a GPER antagonist, and tamoxifen and fulvestrant inhibit ER function through different mechanisms [46,47]; these three drugs are referred to here as EAIs. In this study, we assessed the effects of each EAI or combinations thereof on E2-dependent *S100A8* upregulation in SOECs (Fig 4C). *S100A8* expression was significantly lower in the -E2 group than in the E2 control group (p < 0.01); significantly lower (p < 0.05) in all inhibitor groups than in the E2 group, except for the G-15 + E2 group (*S100A8* mRNA expression was lower than in the E2 control group, but the difference was not significant); and lowest in the tamoxifen group. Tamoxifen downregulates *S100A8* and *S100A9* expression in breast cancer tissues [39]. Our current results indicated that E2 treatment for 7 h upregulated *S100A8* in SOECs, involving both ERs and GPERs; however, the ER-mediated genomic pathway may play a dominant role in *S100A8* upregulation by E2.

### Changes in cytokine levels in SOECs after knocking down the *S100A8* gene

**Fig 5:**
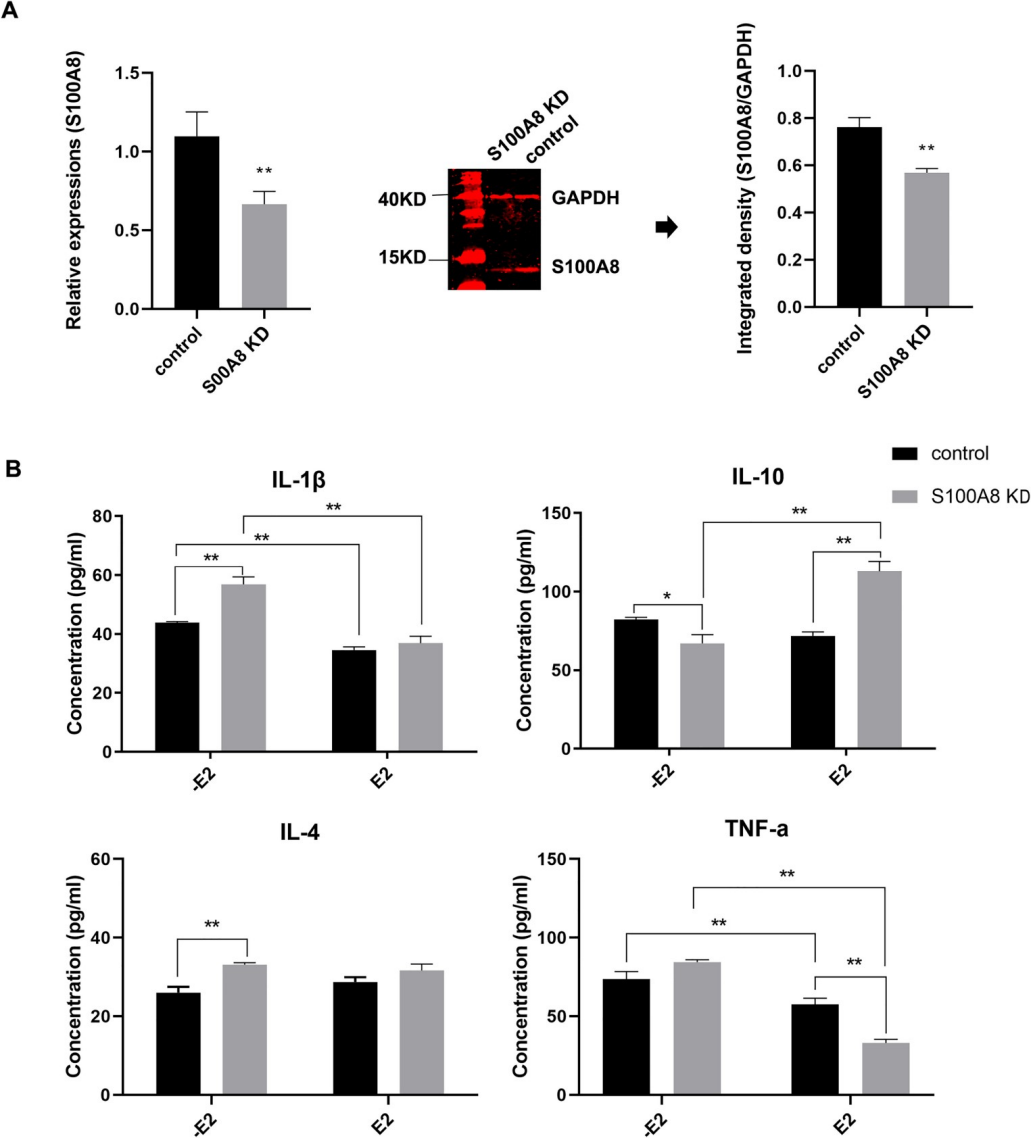
Effects of knocking down the S100A8 gene in SEOCs on cytokine levels. (A) *S100A8* mRNA and protein expression levels in SEOCs were detected by q-PCR and WB analysis with and without (control) *S100A8* gene knockdown. ** (p < 0.01) represents significant difference between the S100A8 knockdown group and the control group. (B) Cytokine (IL-1, IL-10, IL-4, and TNF-α) level changes in the presence or absence of E2 were assessed by ELISA, both with and without (control) S100A8 gene knockdown. * (p < 0.05) and ** (p < 0.01) represent significant difference between two groups.

Knocking out/down the *S100A8* gene and detecting the changes in cytokine levels is helpful for understanding the influence of E2-dependent *S100A8* regulation on the immune environment of the oviduct mucosa. *S100A8* mRNA and protein expression levels in SEOCs were detected 48 h after CRISPR plasmid transfection. Although *S100A8* was not completely knocked out, *S100A8* mRNA and protein expression was significantly lower than that in the control plasmid group (p < 0.01; Fig 5A).

ELISA analysis was performed to study changes in IL-1β, IL-10, IL-4, and TNF-α levels in SOECs after *S100A8* gene knockdown (Fig 5B). Compared with those in the control plasmid group, IL-1β and IL-4 levels in the *S100A8*-knockdown SEOCs were significantly increased (p < 0.01), IL-10 levels were significantly decreased (p < 0.05), and TNF-α levels were not significantly different. IL-4 can promote the expression of several microRNAs related to inflammation, while IL-10 is a Toll-like, receptor 2-dependent, anti-inflammatory factor [48–50]. The nasal inhalation of *S100A8* inhibits lipopolysaccharide-mediated damage in pneumonia through IL-10 and elicits a similar effect to that of dexamethasone [30,31]. These findings suggest that *S100A8* downregulation can cause an excessive immune response, and that *S100A8* helps maintain the immune homeostasis of the oviduct mucosa.

Treating SOECs with 10^−8^ M E2 for 7 h reduced IL-1β and TNF-α levels and increased IL-10 levels in the *S100A8*-knockdown group, which can correct the cytokine imbalance caused by *S100A8* knockdown. In E2-treated SEOCs, *S100A8* knockdown significantly increased IL-10 levels (p < 0.01) compared with those in the control plasmid group. *S100A8* knockdown may cause excessive inflammation, and if E2 cannot upregulate *S100A8*, it can compensate for the effect of *S100A8* knockdown by upregulating IL-10 expression [51–53], which can then correct the dysregulated expression of IL-1β and TNF-α. *S100A8* may play a similar function to IL-10 in regulating the immune homeostasis of the oviduct mucosa induced by estrogen.

## Conclusions

Estrogen affects the immune function of the oviduct mucosa and can dynamically regulate *S100A8* expression in SOECs. Although we tested few chemokines, it can be speculated that reduced *S100A8* expression will cause an excessive immune response, and that *S100A8* is beneficial for maintaining the immune homeostasis of the oviduct mucosa. *S100A8* can potentially serve as a new target for regulating oviduct mucosal dysfunction. In the next study, we hope to further explore how the overexpression of *S100A8* affects the immune function of the oviduct mucosa in the inflammation model.

## Supporting information

S1 Fig S100A8 expression under different EAIs concentration (q-PCR). (TIF)

S1 Table S100A8 expression under E2 treament (5-8h) q-PCR. (XLSX)

S2 Table S100A8 expression under E2 treatment (different concentration) q-PCR. (XLSX)

S3 Table S100A8 expression under EAIs treatment_q-PCR. (XLSX)

S4 Table S100A8 expression under EAIs treatment_WB. (XLSX)

S5 Table S100A8 KD_q-PCR. (XLSX)

S6 Table S100A8 KD WB. (XLSX)

S7 Table S100A8 KO_Immune factor. (XLSX)

S8 Table Expression of five genes (FPKM)_RNA-Seq. (XLSX)

S9 Table Expression of five genes (Relative expression)_q-PCR. (XLSX)

S1 Raw image WB images. (PDF)

S2 Raw image Immunofluorescence images. (PDF)
