# Supplementary figures and images for: S100A8 expression in oviduct mucosal epithelial cells is regulated by estrogen and affects mucosal immune homeostasis
Source: PLoS One. 2021 Nov 18;16(11):e0260188. doi: 10.1371/journal.pone.0260188 (PMC8601440; doi:10.1371/journal.pone.0260188)

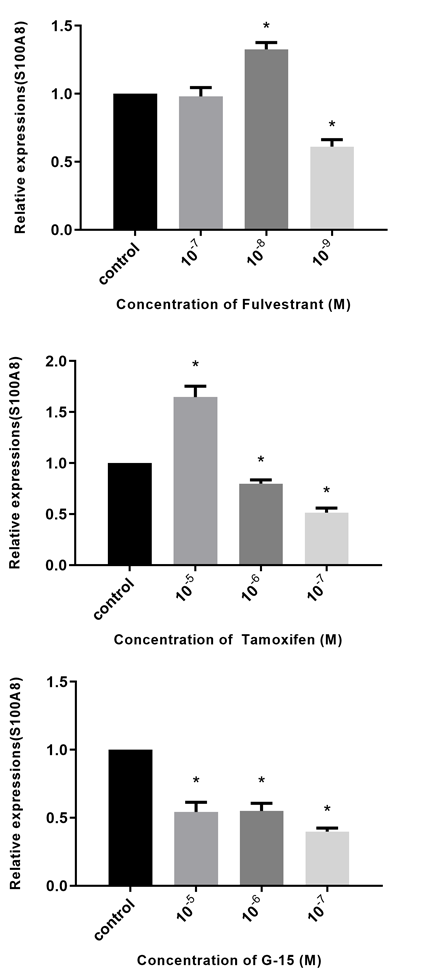

Supplement: S1 Fig — (TIF) [file pone.0260188.s001.tif]

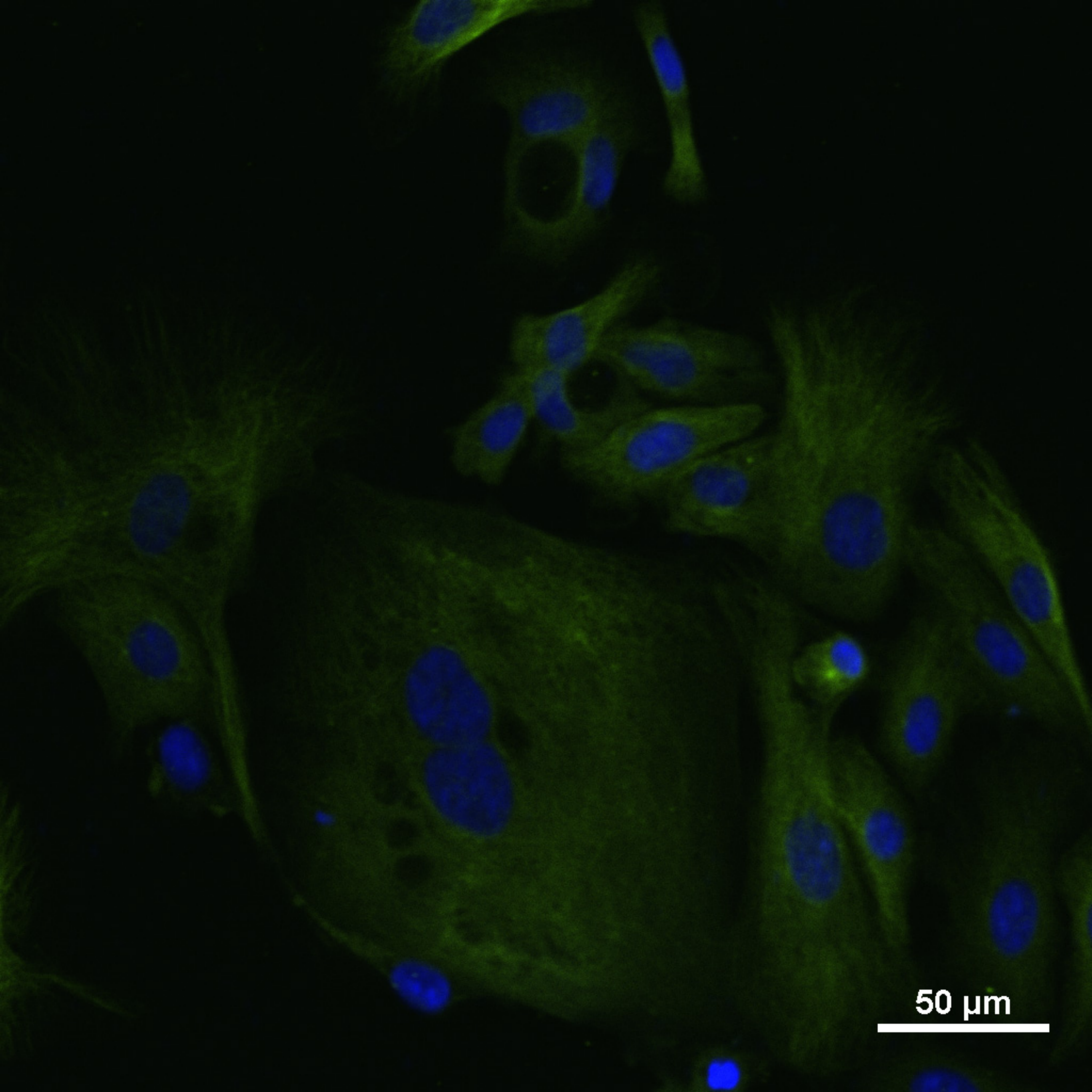

50  $\mu\text{m}$

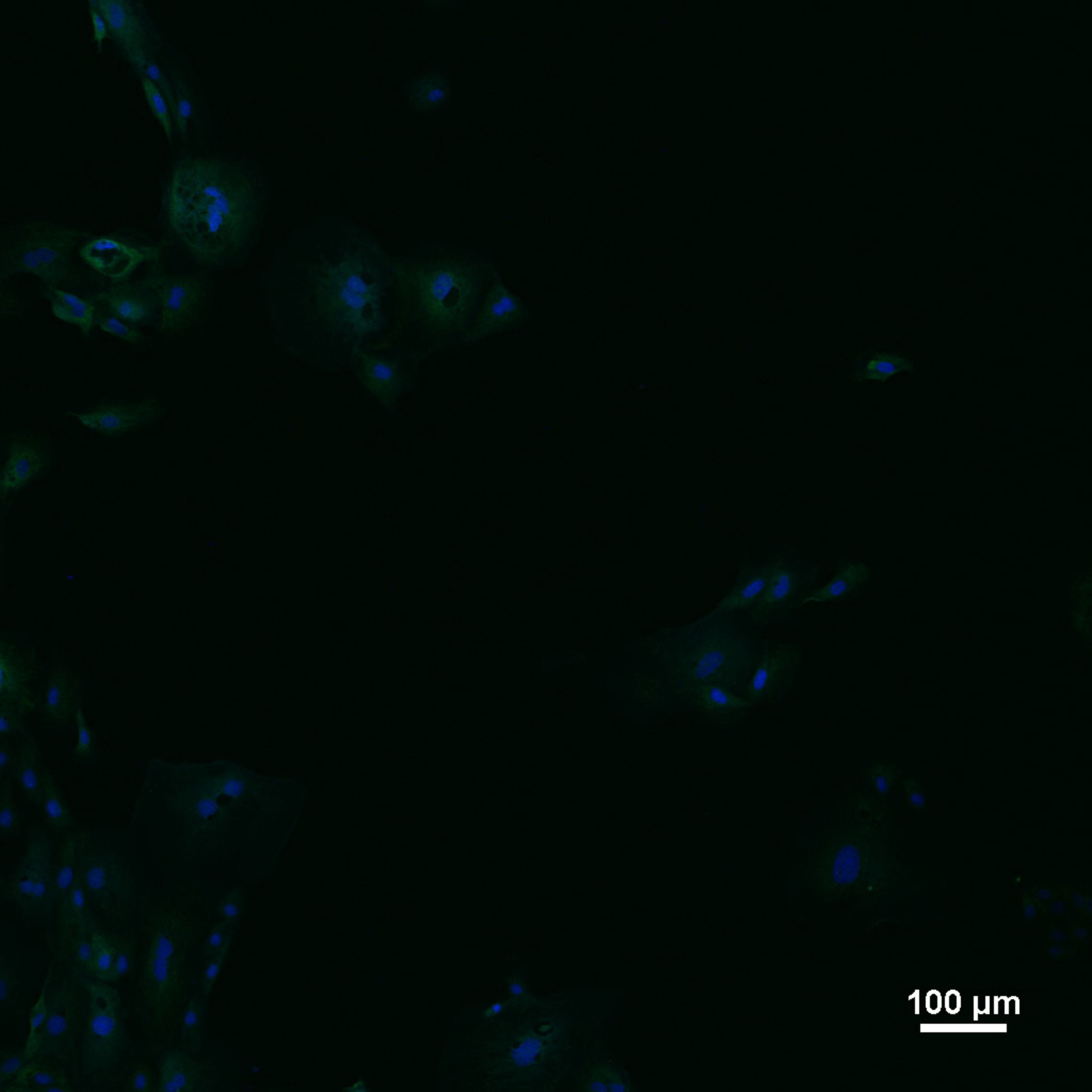

100  $\mu\text{m}$

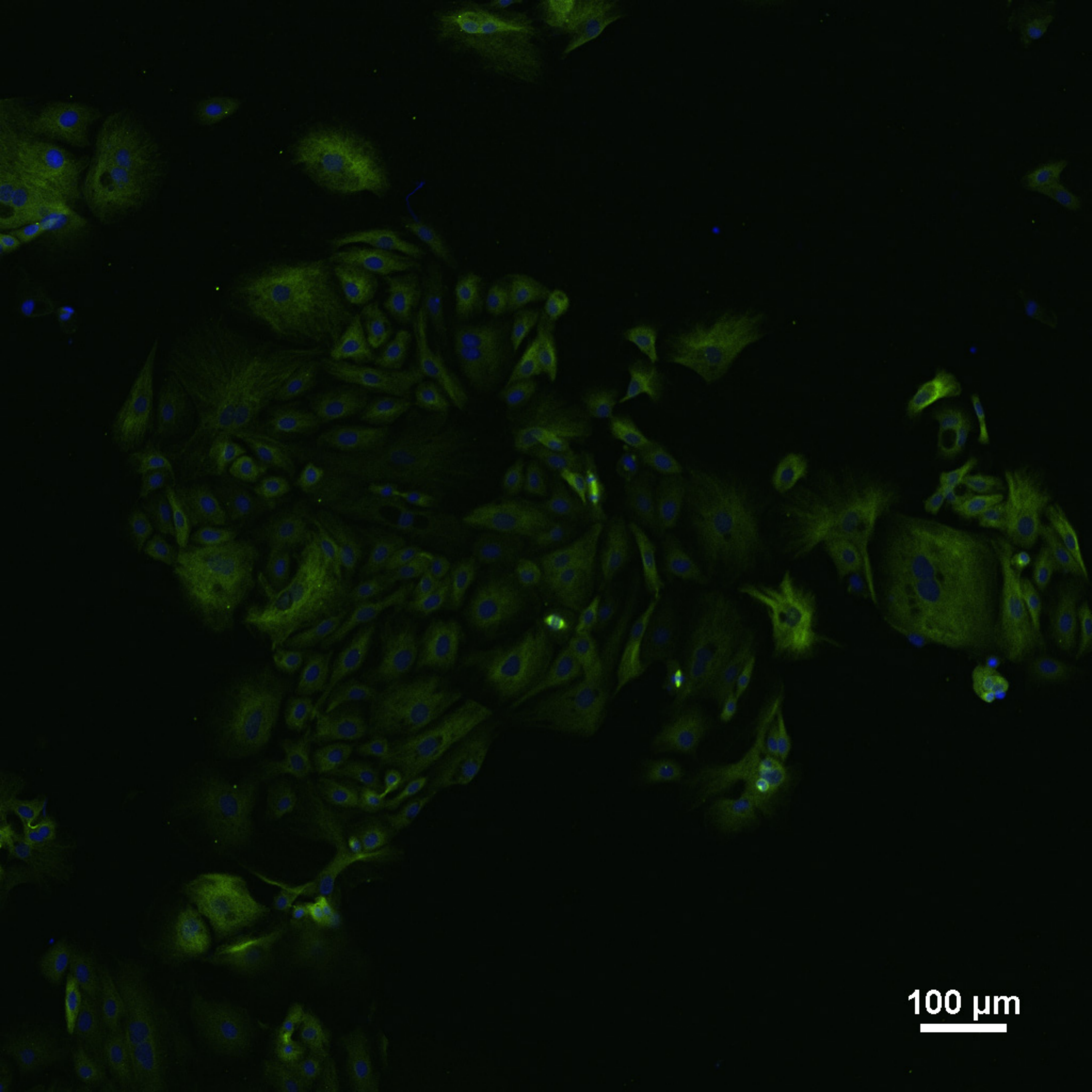

100  $\mu\text{m}$

Supplement: S2 Raw image — (PDF) [file pone.0260188.s012.pdf]
