# Supplementary material for: S100A8 expression in oviduct mucosal epithelial cells is regulated by estrogen and affects mucosal immune homeostasis
Source: PLoS One. 2021 Nov 18;16(11):e0260188. doi: 10.1371/journal.pone.0260188 (PMC8601440; doi:10.1371/journal.pone.0260188)
Supplement: S1 Raw image — (PDF) [file pone.0260188.s011.pdf]

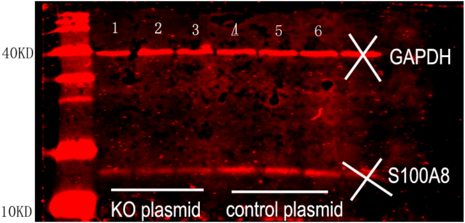

Fluorescent gel imaging system

figure 5-A was generated from lanes 3 and 4 of this original image

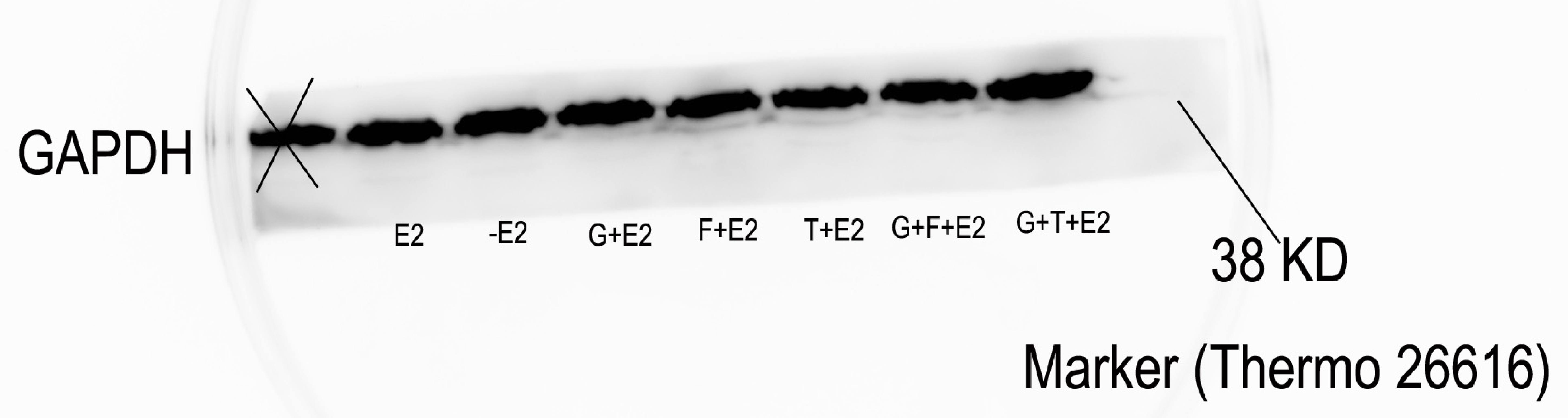

The marker is not seen under the UV light of Gel imaging system,

the bands in this images are used for figure 4-C analysis

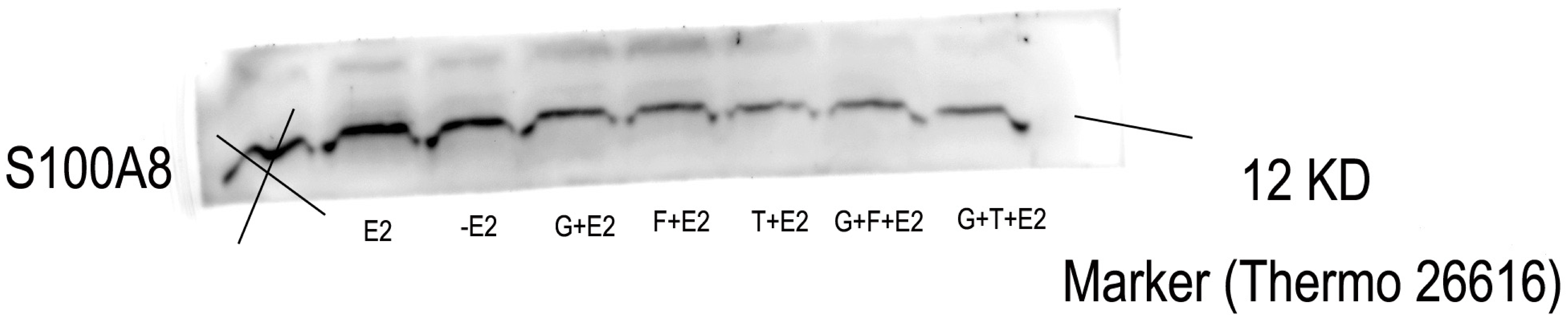

The marker is not seen under the UV light of Gel imaging system,

the bands in this images are used for figure 4-C analysis

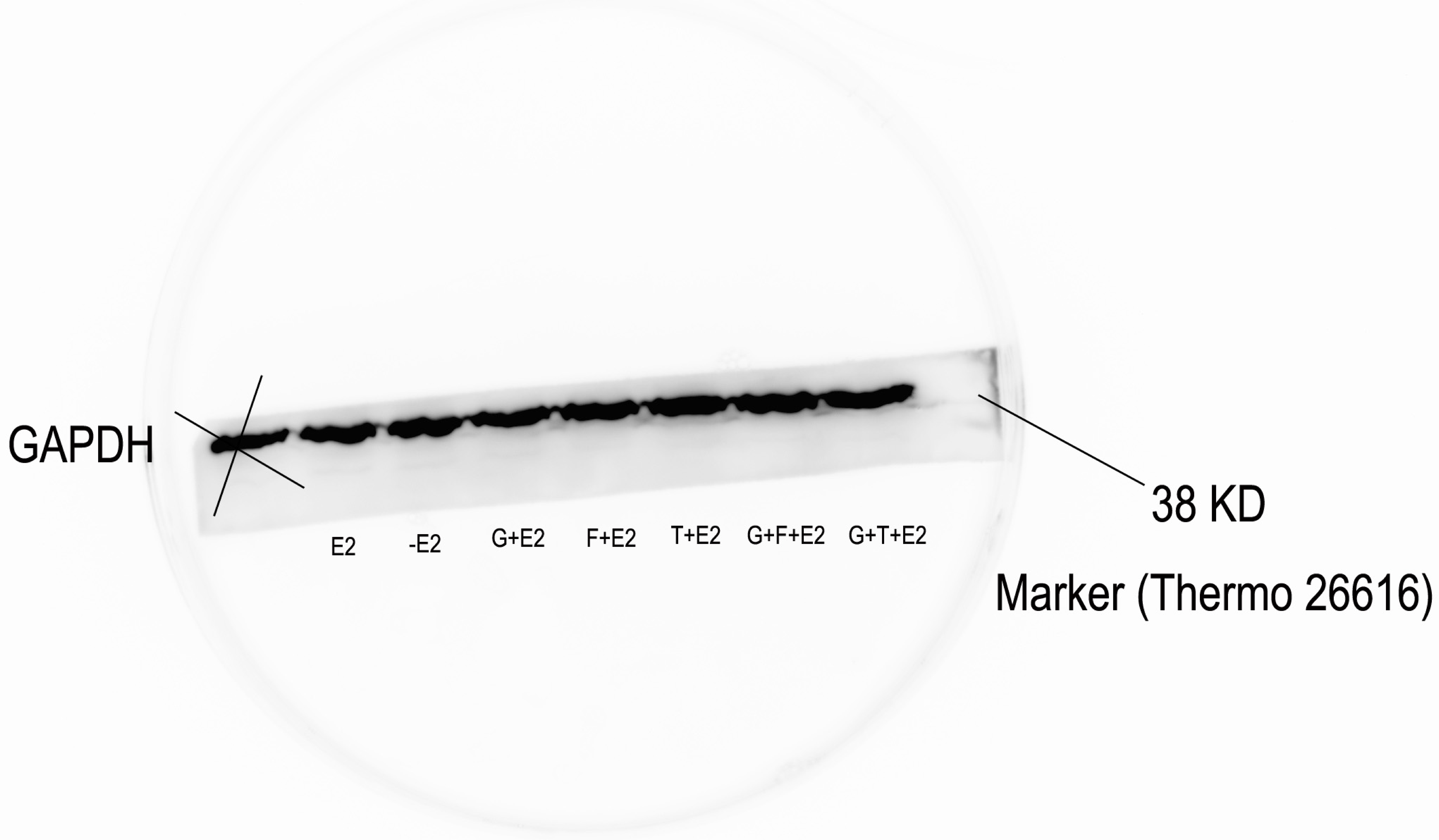

The marker is not seen under the UV light of Gel imaging system,

the bands in this images are used for figure 4-C analysis

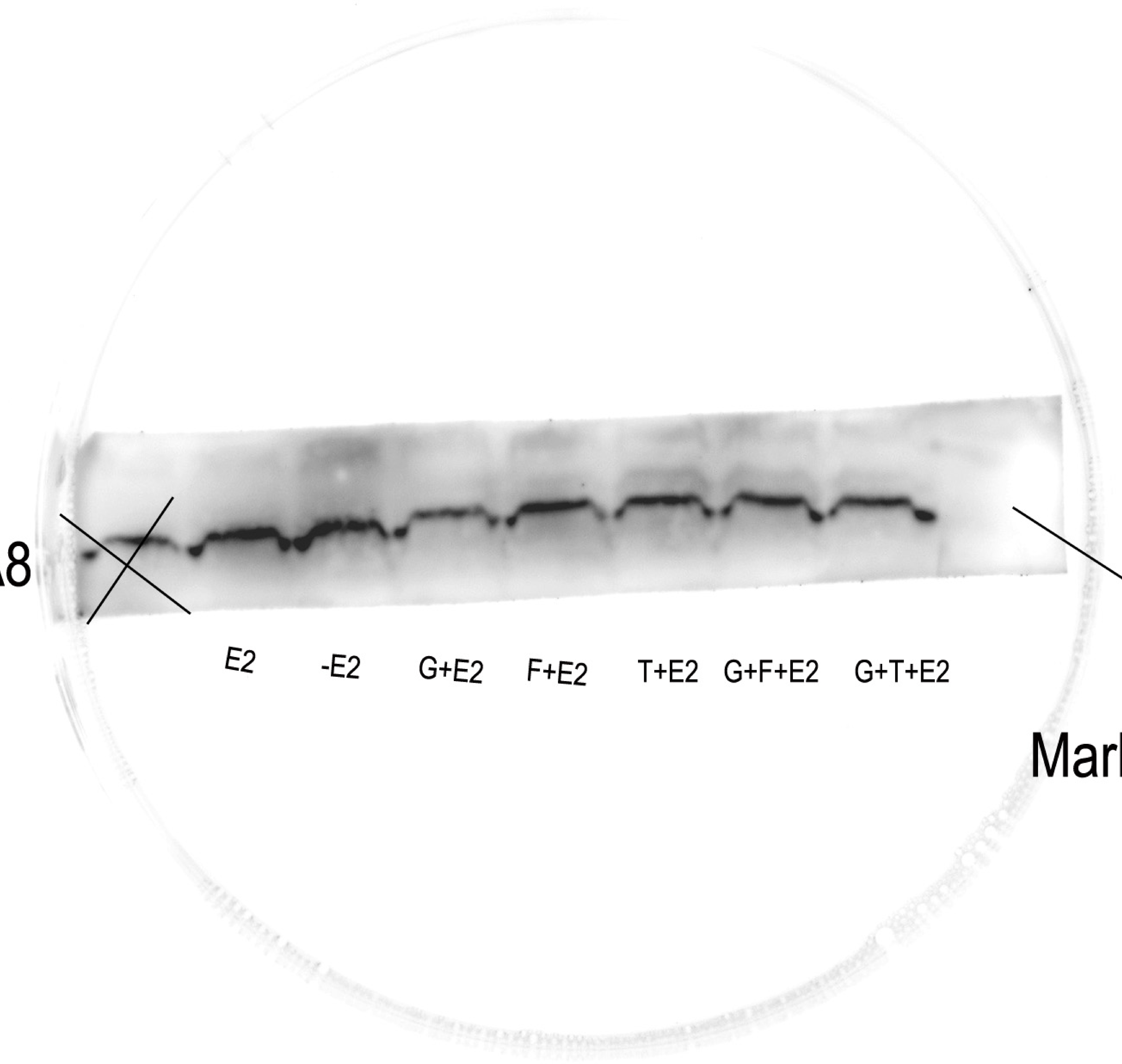

The marker is not seen under the UV light of Gel imaging system,

the bands in this images are used for figure 4-C analysis

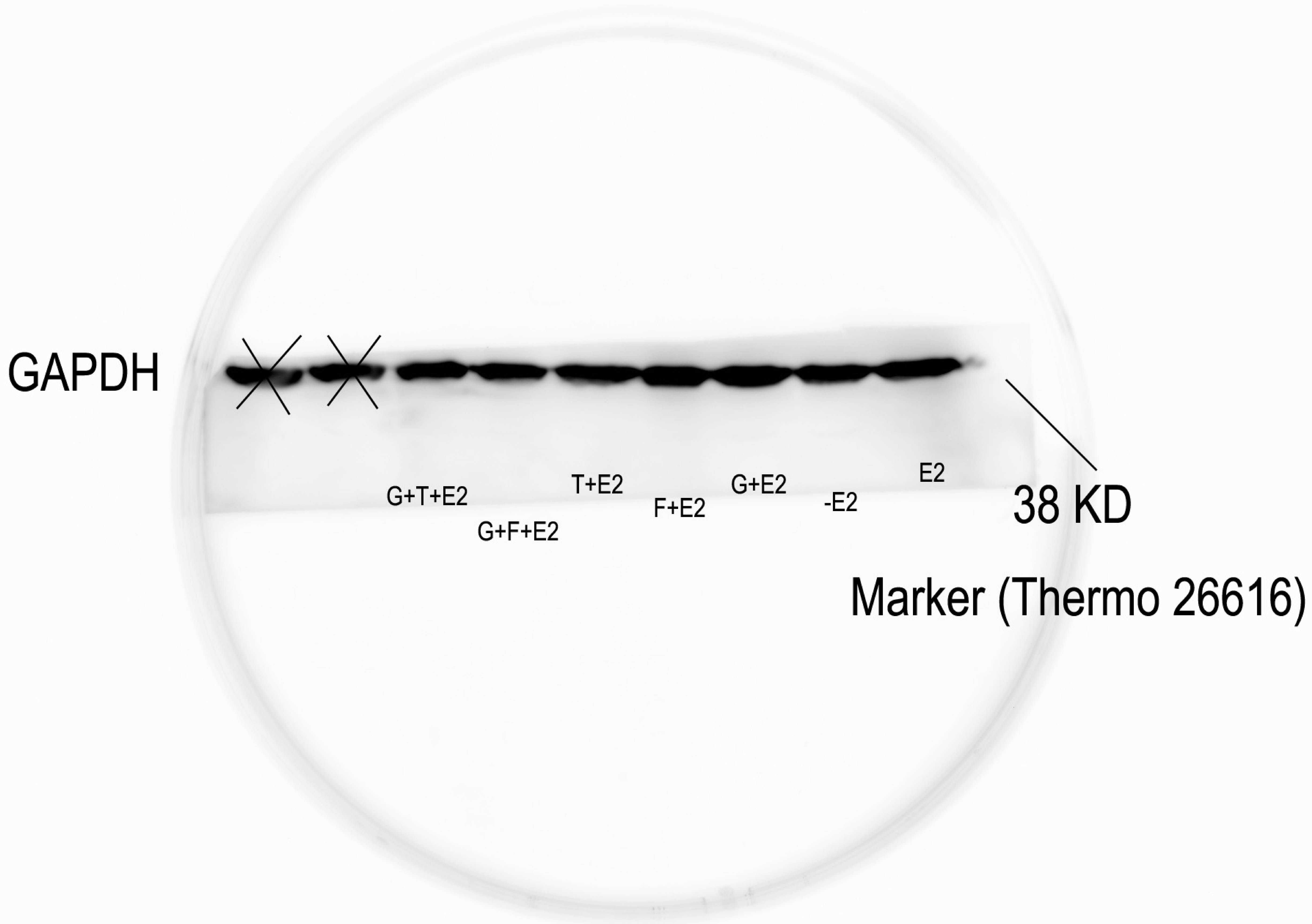

The marker is not seen under the UV light of Gel imaging system (chemiluminescence), figure 4-C was generated from this original image

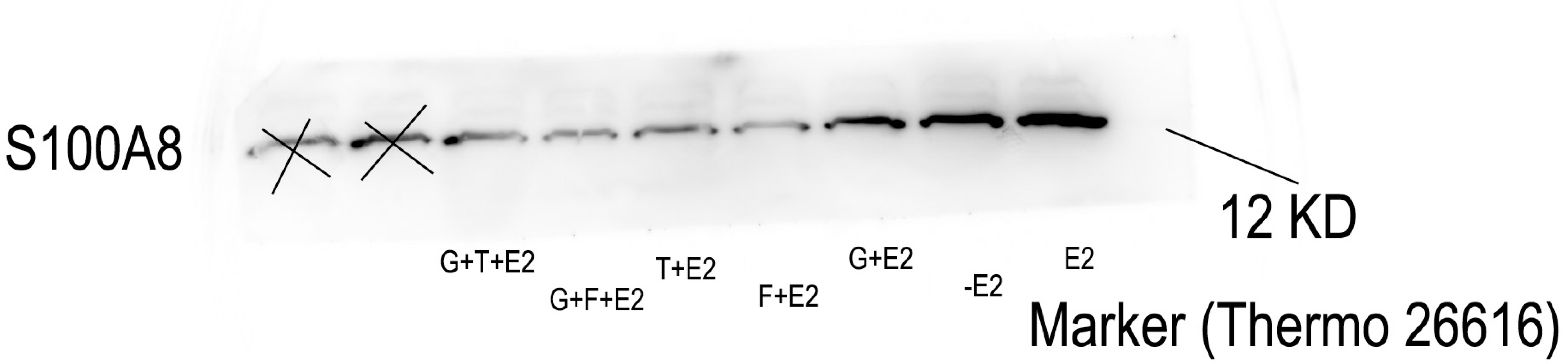

The marker is not seen under the UV light of Gel imaging system,

figure 4-C was generated from this original image
